# Primary School Students’ Online Learning During Coronavirus Disease 2019: Factors Associated With Satisfaction, Perceived Effectiveness, and Preference

**DOI:** 10.3389/fpsyg.2022.784826

**Published:** 2022-03-16

**Authors:** Xiaoxiang Zheng, Dexing Zhang, Elsa Ngar Sze Lau, Zijun Xu, Zihuang Zhang, Phoenix Kit Han Mo, Xue Yang, Eva Chui Wa Mak, Samuel Y. S. Wong

**Affiliations:** ^1^The Jockey Club School of Public Health and Primary Care, Chinese University of Hong Kong, Shatin, Hong Kong SAR, China; ^2^Department of Special Education and Counselling, The Education University of Hong Kong, Tai Po, Hong Kong SAR, China; ^3^Schulich School of Medicine and Dentistry, University of Western Ontario, London, ON, Canada

**Keywords:** online learning, happiness, parent–child relationship, teacher–student interaction, primary school students, COVID-19

## Abstract

Emergency online education has been adopted worldwide due to coronavirus disease 2019 (COVID-19) pandemic. Prior research regarding online learning predominantly focused on the perception of parents, teachers, and students in tertiary education, while younger children’s perspectives have rarely been examined. This study investigated how family, school, and individual factors would be associated with primary school students’ satisfaction, perceived effectiveness, and preference in online learning during COVID-19. A convenient sample of 781 Hong Kong students completed an anonymous online survey from June to October 2020. Logistic regression was conducted for 13 potential factors. Results indicated that only 57% of students were satisfied with their schools’ online learning arrangement and 49.6% regarded the online learning as an effective learning mode. Only 12.8% of students preferred online learning, while 67.2% of students preferred in-person schooling. Multiple analyses suggested that teacher–student interaction during online classes was positively associated with students’ satisfaction, perceived effectiveness, and preferences in online learning. Compared to grades 1–2 students, grades 3–6 students perceived more effectiveness and would prefer online learning. Happier schools were more likely to deliver satisfying and effective online education. Students who reported less happiness at school would prefer online learning, and students who reported less happiness at home would be less satisfied with online learning and reflected lower effectiveness. Teachers are encouraged to deliver more meaningful interactions to students and offer extra support to younger children during online classes. Primary schools and parents are encouraged to create a healthy and pleasant learning environment for children. The government may consider building up happy schools in the long run. The study findings are instrumental for policymakers, institutions, educators, and researchers in designing online education mechanisms.

## Introduction

The worldwide education systems were severely affected due to a series of consequent infection control measures responding to the outbreak of coronavirus disease 2019 (COVID-19), with up to two-thirds of an academic year lost on average worldwide due to school closure (Cucinotta and Vanelli, 2020). In Hong Kong, the first COVID-19 case was confirmed on January 23, 2020 (Wong et al., 2020) and the Education Bureau (EDB) has announced school closure and class suspension amid the several consequent waves of the epidemic (The Government of the Hong Kong Special Administrative Region, 2020). Although schools would sometimes resume regular operation during the lockdown, most schools were required to arrange just a limited number of students to return to school everyday for face-to-face classes or activities.

To accommodate the unexpected disturbances, educational institutions had to abruptly shift to online teaching to ensure the Students’ continuous learning in this pandemic era (Aboagye et al., 2021). However, a smooth shift could be challenging. Students have encountered lots of obstacles, such as lack of access to devices for online learning (Almanthari et al., 2020; Dube, 2020), unstable internet connectivity (Rotas and Cahapay, 2020), lack of technical know-how of devices (Owusu-Fordjour et al., 2020), schools’ inexperience in offering online education (Wodon, 2020), family’s financial unpreparedness (Agormedah et al., 2020), lack of parental support (Owusu-Fordjour et al., 2020), and feeling bored due to lack of interpersonal communication (Irawan et al., 2020). Likewise, parents expressed their worries about children’s increased screen time, more exposure to harmful content over the Internet, reduced physical activities, and lack of socializing (Harjule et al., 2021). Moreover, teachers faced challenges in implementing online teaching due to insufficient training with digital tools, absence of constant contact with students to monitor their study routine, and lack of support and assistance from parents (Shamir-Inbal and Blau, 2021).

The obstacles above alerted us that students could be vulnerable to receiving satisfying and effective online education during COVID-19. The situation could be even worse for primary school students, who are still developing their self-regulation and attention control skills and are incompetent to handle technological problems and other emergencies independently (Gallagher and Cottingham, 2020) compared to students in secondary and tertiary educations.

Therefore, special attention should be paid to the primary school Students’ online learning amid COVID-19 pandemic, with their demands, difficulties encountered, and expectations deeply understood. As for younger children, learning at home means parental support is crucial. The abrupt shift to online learning was challenging and an issue of concern owing to the lack of sufficient support offered to the parents and this may facilitate parental burnout, which would passively impact children’s well-being and online learning during COVID-19 pandemic (Griffith, 2020). So, it is vital to investigate young children’s relationships with their parents (Sheehan et al., 2019) and their happiness level at home (Fidan, 2021) when they are receiving online education. Thus, we examined how children’s satisfaction with parents and happiness at home, as family factors, would be associated with their online learning. Besides, as the efficacy of online education relied on the school resources available (Tran et al., 2020), school factors were also examined, including teacher–student interaction (Alqurashi, 2019), school’s happiness index rated by students (Cote, 2006), and Students’ happiness level at school (Mauro and Machell, 2019). In addition, children’s individual factors were another category worthy of exploring as prior research has substantiated that loneliness (ValÅs, 1999), sleep (Peigneux et al., 2001; Al-Sharman and Siengsukon, 2013), self-awareness (Steiner, 2014), academic performance (Zeegers, 2004), and mobility in schools (Sorin and Iloste, 2006) would significantly influence Students’ learning. Therefore, correlations between online learning and Students’ individual factors, including self-perceived loneliness, sleep time, satisfaction with self-performance, perceived academic performance, and intention of school transfer, were demonstrated in this study.

To summarize, from primary school Students’ perspectives, this study explored 10 potential factors from three aspects: (a) Student’s family factors (satisfaction with parents and happiness level at home); (b) Student’s school factors (happiness level at school, school’s happiness index, and teacher–student interaction); and (c) Student’s individual factors (self-perceived loneliness, sleep time, satisfaction with self-performance, perceived academic performance, and intention of school transfer), to investigate how these factors would influence Students’ perception of online learning during COVID-19 pandemic.

This study aimed to provide policymakers, institutions, educators, and researchers a deeper insight into younger children’s experience in receiving online learning in this unexpected crisis, which was rarely explored as prior research predominantly focused on the views of parents, teachers, and students in tertiary education. The findings of this study can serve as instrumental references and inspirations in refining pedagogical designs to better adapt primary school students to the online learning practice during COVID-19 or under future tendencies and unexpected crises.

## Materials and Methods

### Participants

As primary schools were periodically closed as instructed by the EDB during this study period, convenient sampling (Lavrakas, 2008) was adopted to search prospective primary schools. The team sent out invitation emails to 165 randomly selected primary schools of four types [i.e., government, aided, the Direct Subsidy Scheme (DSS), and private]^1^ with at least three reminders and follow-up telephone calls. Finally, six primary schools (3 aided, 2 DSS, and 1 private) agreed to participate in this study. These six schools then send out invitation emails with the online survey link to students and parents to participate voluntarily and anonymously.

### Instrument

The questionnaire was developed by an expert panel consisting of a group of researchers and practitioners in public health, education, and journalism. Initially, a Chinese version of the questionnaire was preliminarily developed. Each panel member independently reviewed the questionnaire and provided their comments. Several rounds of discussion and revision were conducted until the panel finally agreed on a relatively shorter version that took only 5–10 min to complete. Considering the lower literacy skills in younger pupils who required cognitive scaffolding in filling in the questionnaire (Tomasik et al., 2020), audios for all the questions and answers were provided in the questionnaire for primary 1–3 students. Colorful pictures and emojis were also embedded to facilitate their understanding. Four bilingual researchers experienced in public health and education worked together to translate the Chinese questionnaire into English to accommodate Hong Kong’s bilingual educational needs. The English version had gone through several rounds of revision and proofreading to guarantee that all the questions and answer options had equivalent meaning as those in the Chinese version. A pilot study was administered to 45 primary school students with at least five students from each of the six grades. Good acceptability was indicated for the survey, with minor revisions made based on feedback collected in the pilot.

The questionnaire was generated *via* a widely used online survey software (QuestionPro). Participants could fill in it *via* different electronic devices, such as computers, tablets, and smartphones. Parents were invited to aid their children. Participants’ consent would be sought before starting the questionnaire. A click of “Yes” would lead to starting the questionnaire, while a click of “No” would lead to a termination of the questionnaire. The survey was conducted from June to October 2020.

### Measurement

#### Study Outcomes

Students’ satisfaction, perceived effectiveness, and preference in online learning using the Likert-type scale (Joshi et al., 2015) constitute the three outcomes. Satisfaction was measured by asking “During the period of school suspension, do you like the arrangement made by your school regarding learning at home?,” with five answer options categorized as “like a lot,” “quite like,” “average,” “do not quite like,” and “dislike a lot.” Perceived effectiveness was measured by asking “What do you think about the effect of learning at home?,” with five answer options as “very good,” “quite good,” “average,” “quite bad,” and “very bad.” Preference was measured by asking “Do you prefer to study online or at school?,” with three answer options as “prefer to study online,” “prefer to study at school,” and “same.”

#### Independent Variables

Thirteen items serve as the independent variables. Demographic characteristics were collected, including the type of primary school, grade of study, and gender. Ten items measured the Student’s loneliness, sleep time, happiness at school, happiness at home, satisfaction with parents, satisfaction with self-awareness, academic performance, the intention of school transfer, teacher–student interaction during online learning, and school’s happiness index rated by the student. Loneliness was measured using the UCLA Three-Item Loneliness Scale (Hughes et al., 2004). The other nine items were devised using the Likert-type scale (Joshi et al., 2015). Sleep time was measured by asking “Do you have sufficient sleep?” with three answer options as “sufficient,” “not sufficient,” and “very insufficient.” Satisfaction with parents was measured by asking “Overall speaking, what is your degree of satisfaction with your father and mother?” with five answer options as “very satisfied,” “satisfied,” “half-and-half,” “not quite satisfied,” and “very dissatisfied.” Satisfaction with self-performance was measured by asking “Do you feel satisfied with your performance in various aspects?” with five answer options as “very satisfied,” “satisfied,” “half-and-half,” “not quite satisfied,” and “very dissatisfied.” Perceived academic performance was measured by asking “In your opinion, your academic results are,” with five answer options as “the best,” “good,” “moderate,” “bad,” “the worst.” The intention of school transfer was measured by asking “Do you want to study at another school?,” with five answer options as “don’t want at all,” “don’t quite want,” “average,” “slightly want,” and “want very much.” Teacher–student interaction was measured by asking “Do teachers interact with you when you study online at home?,” with five answer options as “always,” “often,” “average,” “seldom,” and “rarely.” As for happiness at school, happiness at home, and school’s happiness index, participants were asked to rate from 0 to 10, with 0 representing the least happy and 10 representing the happiest. Happiness at school was measured by asking “If you are to give a mark, the mark representing the happiness you feel at school is.” Happiness at home was measured by asking “If you are to give a mark, the mark representing the happiness you feel at home is.” School’s happiness index was measured by asking “If you are to give a mark to your school’s happiness index, you will give it.”

### Statistical Analysis

Frequency and percentage of the categorical variables and mean and SD of the continuous variables were presented for data description. Four principal assumptions of linear regression were tested, but the linearity and homoscedasticity assumptions were violated, so a linear model cannot be used (Alexopoulos, 2010). The Likert-type scale used for dependent variables presented ordinal data and categorical data (Robertson, 2012). The five-point or three-point scales of the variables were compressed to lesser points to ensure that each cross-tabulation group has sufficient number, so that valid analysis results could be presented. The assumptions of logistic regression were tested (Healy, 2006) and found satisfied. The dependent variables were dichotomous and no outlier data were found that could distort the outcome and accuracy of the model. Therefore, logistic regression was used in analyzing the data.

The univariate logistic regression analysis was applied to explore the association between each independent variable and each dependent variable and multiple regression analysis was applied to examine the association between all the independent variables and each dependent variable. Adjusted odds ratios (AORs) and their 95% CIs were presented. Statistical significance was considered when *p*-values were < 0.05 (two sides). Statistical analyses were performed using the statistical package IBM SPSS Statistics (version 26).

## Results

A total of 781 students completed the survey, with 60.5% from aided schools and 39.5% from the DSS and private schools. About 35.2% of students were studying in grades 1–2, 28.8% of students were studying in grades 3–4, and 36.0% of students were studying in grades 5–6. About 67.2% of students were male and 32.8% of students were female.

Students’ responses to the questionnaire and their demographic characteristics are given in Table 1. Half of the students (50.1%) reported that teachers often or always interacted with them during online learning. Less than three-fifths (57%) were satisfied with their schools’ online learning arrangement. Nearly half (49.6%) reflected the effect of online learning as good or very good. About one-eighth students (12.8%) preferred online learning and the rest preferred in-person schooling (67.2%) or did not indicate any preference (20.0%).

**TABLE 1:** Students’ demographic characteristics and responses to the survey items (*N* = 781).

Item	*N*	%
**Type of primary school*^a^***		
Aided	460	60.5
Direct Subsidy Scheme (DSS)/Private	300	39.5
**Grade of study**		
Low grade (P1–P2)	275	35.2
Middle grade (P3–P4)	225	28.8
High grade (P5–P6)	281	36.0
**Gender**		
Male	525	67.2
Female	256	32.8
**Self-perceived loneliness**		
Not lonely	613	78.5
Lonely	168	21.5
**Sleep time**		
Insufficient	277	35.5
Sufficient	504	64.5
**Happiness at school and home**		
Happier both at school and home (9–10)	329	42.1
Less happy both at school and home (0–8)	210	26.9
Happier at home (9–10) while less happy at school (0–8)	149	19.1
Happier at school (9–10) while less happy at home (0–8)	93	11.9
**School’s happiness index** (0–10) (*Mean, SD*)*^b^*	8.26	1.83
**Satisfaction with parents**		
Very dissatisfied/Not quite satisfied	35	4.5
Half-and-half	116	14.9
Satisfied/Very satisfied	630	80.7
**Satisfaction with self-performance**		
Very dissatisfied/Not quite satisfied	70	9.0
Half-and-half	226	28.9
Satisfied/Very satisfied	485	62.1
**Perceived academic performance**		
The worst/Bad	74	9.5
Moderate	309	39.6
Good/The best	398	51.0
**Intention of school transfer**		
Strongly agree/Agree	26	3.3
Average	97	12.4
Disagree/Strongly disagree	658	84.3
**Do teachers interact with you when you study online at home?**		
Rarely/Seldom	133	17.0
Average	257	32.9
Often/Always	391	50.1
**Do you like the arrangement made by your school regarding learning at home?**		
Dislike a lot/Don’t quite like/Average	336	43.0
Quite like/Like a lot	445	57.0
**What do you think about the effect of learning at home?**		
Very bad/Bad/Average	394	50.4
Good/Very good	387	49.6
**Do you prefer to study online or at school?**		
Study online	100	12.8
Study at school	525	67.2
No preference	156	20.0

*^a^21 Missing cases.*

*^b^The results are presented by mean and standard deviation (SD).*

### Satisfaction in Online Learning Arrangement

In the univariate model, satisfaction with the online learning arrangement was significantly associated with studying in the DSS or private schools, being male, rating higher happiness indexes for schools, being satisfied with parents and self-performance, gaining better perceived academic performance, having no intention of school transfer, and receiving more teacher–student interaction during online learning. However, students who felt less happy both at the school and home and those who felt less happy either at school or home were less likely to be satisfied with the online learning arrangement than those who felt happy both at school and home (*p* < 0.05, Table 2).

**TABLE 2:** The univariate regression results of factors associated with Students’ views on online learning.^

Items	Quite like/Like a lot the		Perceived good/very		Prefer to study	
	online learning arrangement*^a^*		good effectiveness*^b^*		online*^c^*	
	OR (95%CI)	*P*-value	OR (95%CI)	*P*-value	OR (95%CI)	*P*-value
**Type of primary school**						
Aided	Ref		Ref		Ref	
Direct Subsidy Scheme (DSS)/Private	1.50 (1.12, 2.02)	0.007**	1.65 (1.23, 2.21)	0.001**	0.88 (0.56, 1.36)	0.552
**Grade**						
Grade 1–2	Ref		Ref		Ref	
Grade 3–4	1.05 (0.73, 1.50)	0.805	1.13 (0.79, 1.60)	0.506	3.41 (1.66, 7.02)	0.001**
Grade 5–6	0.78 (0.56, 1.09)	0.139	0.92 (0.66, 1.28)	0.616	6.66 (3.42, 12.96)	<0.001**
**Gender**						
Female	Ref		Ref		Ref	
Male	1.42 (1.05, 1.92)	0.022*	1.41 (1.05, 1.91)	0.024*	1.16 (0.73, 1.83)	0.526
**Self-perceived loneliness**						
Not lonely	Ref		Ref		Ref	
Lonely	0.74 (0.53, 1.05)	0.088	0.73 (0.52, 1.03)	0.075	2.34 (1.49, 3.67)	<0.001**
**Sleep time**						
Insufficient	Ref		Ref		Ref	
Sufficient	1.20 (0.89, 1.61)	0.237	1.08 (0.80, 1.44)	0.626	0.63 (0.41, 0.97)	0.034*
**Happiness at school and home**						
Happy both at school and home (9–10)	Ref		Ref		Ref	
Less happy both at school and home (0–8)	0.35 (0.24, 0.50)	<0.001**	0.39 (0.27, 0.55)	<0.001**	4.75 (2.59, 8.71)	<0.001**
Happy at home (9–10) while less happy at school (0–8)	0.65 (0.44, 0.97)	0.034**	0.68 (0.46, 1.00)	0.052	7.68 (4.15, 14.22)	<0.001**
Happy at school (9–10) while less happy at home (0–8)	0.49 (0.30, 0.78)	0.002**	0.46 (0.29, 0.74)	0.001**	0.21 (0.03, 1.63)	0.136
**School’s happiness index**						
0–8	Ref		Ref		Ref	
9–10	2.24 (1.67, 2.99)	<0.001**	2.01 (1.51, 2.67)	<0.001**	0.22 (0.13, 0.35)	<0.001**
**Satisfaction with parents**						
Half-and-half/Dissatisfied	Ref		Ref		Ref	
Satisfied	1.54 (1.08, 2.20)	0.017**	1.53 (1.07, 2.19)	0.021*	0.48 (0.30, 0.76)	0.002**
**Satisfaction with self-performance**						
Half-and-half/Dissatisfied	Ref		Ref		Ref	
Satisfied	1.89 (1.41, 2.53)	<0.001**	2.30 (1.71, 3.10)	<0.001**	0.37 (0.24, 0.57)	<0.001**
**Perceived academic performance**						
Moderate/The worst/Bad	Ref		Ref		Ref	
Good/The best	1.89 (1.42, 2.52)	<0.001**	2.44 (1.83, 3.25)	<0.001**	0.43 (0.27, 0.66)	<0.001**
**Intention of school transfer**						
Average/Agree	Ref		Ref		Ref	
Disagree	1.66 (1.13, 2.45)	0.010**	1.53 (1.04, 2.27)	0.032*	0.37 (0.23, 0.60)	<0.001**
**Teacher-student interaction during online learning**						
Rarely/Seldom	Ref		Ref		Ref	
Average	1.98 (1.28, 3.06)	0.002**	1.97 (1.24, 3.11)	0.004**	1.18 (0.6, 2.19)	0.590
Often/Always	4.74 (3.11, 7.22)	<0.001**	4.75 (3.07, 7.36)	<0.001**	0.89 (0.49, 1.61)	0.695

^ * p < 0.05; ** p < 0.01.

*^a^Dislike a lot/Don’t quite like/Average were regarded as reference category.*

*^b^Very bad/Bad/Average were regarded as reference category.*

*^c^Prefer to study at school and the same were regarded as reference category.*

*Bold values indicate statistically significance.*

In the multiple model, more teacher–student interactions during online learning (average: AOR = 2.08, 95% CI: 1.31–3.29, *p* = 0.002; often or always: AOR = 4.11, 95% CI: 2.61–6.48, *p* < 0.001) and higher happiness indexes for schools (AOR = 1.84, 95% CI: 1.14–2.98, *p* = 0.013) remained significantly associated with being satisfied with the online learning arrangement. Students who felt happy at school while less happy at home (AOR = 0.57, 95% CI: 0.34–0.96, *p* = 0.033) remained less likely to be satisfied than those who felt happy both at the school and home (Table 3).

**TABLE 3:** Multiple regression results of factors associated with Students’ views on online learning.

Items	Quite like/Like a lot the		Perceived good/very		Prefer to study	
	online learning arrangement*^a^*		good effectiveness*^b^*		online*^c^*	
	AOR (95%CI)	*P*-value	AOR (95%CI)	*P*-value	AOR (95%CI)	*P*-value
**Type of primary school**						
Aided	Ref	Ref	Ref			
Direct Subsidy Scheme (DSS)/Private	1.41 (0.96, 2.08)	0.084	1.50 (1.02, 2.21)	0.042*	1.37 (0.76, 2.47)	0.298
**Grade**						
Grade 1–2	Ref	Ref	Ref			
Grade 3–4	1.41 (0.92, 2.15)	0.112	2.00 (1.31, 3.06)	0.001**	4.74 (2.20, 10.22)	<0.001**
Grade 5–6	1.36 (0.92, 2.03)	0.127	1.60 (1.08, 2.38)	0.020*	3.77 (1.70, 8.36)	0.001**
**Gender**						
Female	Ref	Ref	Ref			
Male	1.19 (0.81, 1.76)	0.381	1.09 (0.74, 1.62)	0.663	1.57 (0.87, 2.85)	0.136
**Self-perceived loneliness**						
Not lonely	Ref	Ref	Ref			
Lonely	1.38 (0.89, 2.15)	0.152	1.25 (0.80, 1.96)	0.323	1.21 (0.68, 2.15)	0.514
**Sleep time**						
Insufficient	Ref	Ref	Ref			
Sufficient	0.97 (0.69, 1.37)	0.866	0.77 (0.55, 1.09)	0.139	0.80 (0.48, 1.32)	0.380
**Happiness at school and home**						
Happy both at school and home (9–10)	Ref		Ref		Ref	
Less happy both at school and home (0–8)	0.64 (0.37, 1.12)	0.116	0.69 (0.39, 1.21)	0.196	2.17 (0.86, 5.44)	0.099
Happy at home (9–10) while less happy at school (0–8)	1.23 (0.70, 2.16)	0.477	1.21 (0.69, 2.13)	0.516	5.20 (2.19, 12.34)	<0.001**
Happy at school (9–10) while less happy at home (0–8)	0.57 (0.34, 0.96)	0.033*	0.55 (0.33, 0.93)	0.025*	0.13 (0.02, 1.05)	0.056
**School’s happiness index**						
0–8	Ref	Ref	Ref			
9–10	1.84 (1.14, 2.98)	0.013*	1.67 (1.03, 2.71)	0.039*	0.89 (0.41, 1.93)	0.767
**Satisfaction with parents**						
Half-and-half/Dissatisfied	Ref	Ref	Ref			
Satisfied	0.81 (0.53, 1.25)	0.347	0.80 (0.52, 1.25)	0.330	0.53 (0.29, 0.95)	0.033*
**Satisfaction with self-performance**						
Half-and-half/Dissatisfied	Ref	Ref	Ref			
Satisfied	1.07 (0.73, 1.57)	0.734	1.38 (0.94, 2.02)	0.103	0.59 (0.33, 1.04)	0.066
**Perceived academic performance**						
Moderate/The worst/Bad	Ref	Ref	Ref			
Good/The best	1.29 (0.91, 1.85)	0.156	1.76 (1.23, 2.51)	0.002**	0.78 (0.44, 1.37)	0.388
**Intention of school transfer**						
Average/Agree	Ref	Ref	Ref			
Disagree	1.18 (0.75, 1.86)	0.484	1.10 (0.69, 1.76)	0.680	0.74 (0.42, 1.31)	0.302
**Teacher-student interaction during online learning**						
Rarely/Seldom	Ref	Ref	Ref			
Average	2.08 (1.31, 3.29)	0.002**	2.08 (1.28, 3.38)	0.003**	1.51 (0.77, 2.97)	0.231
Often/Always	4.11 (2.61, 6.48)	<0.001**	4.22 (2.62, 6.79)	<0.001**	2.32 (1.17, 4.61)	0.017*

*p < 0.05; **p < 0.01.

*^a^Dislike a lot/Don’t quite like/Average were regarded as reference category; Chi-square value and p-value under Hosmer and Lemeshow Test are 7.064 and 0.530. ^b^Very bad/Bad/Average were regarded as reference category; Chi-square value and p-value under Hosmer and Lemeshow Test are 15.691 and 0.047.*

*^c^Prefer to study at school and the same were regarded as reference category; Chi-square value and p-value under Hosmer and Lemeshow Test are 6.577 and 0.583.*

*Bold values indicate statistically significance.*

### Perceived Effectiveness in Online Learning

In the univariate model, greater online learning effectiveness was significantly correlated with studying in the DSS or private school, being male, rating higher happiness indexes for schools, being satisfied with parents and self-performance, gaining better perceived academic performance, having no intention of school transfer, and receiving more teacher–student interaction during online learning. Also, students who felt less happy both at school and home and those who felt happy at school while less happy at home were less likely to perceive the effectiveness of online learning than those who felt happy both at school and home (Table 2, *p* < 0.05).

In the multiple model, more teacher–student interactions during online learning (average: AOR = 2.08, 95%CI: 1.28–3.38, *p* = 0.003; often or always: AOR = 4.22, 95% CI: 2.62–6.79, *p* < 0.001), higher grade (Grades 3–4: AOR = 2.00, 95% CI: 1.31–3.06, *p* = 0.001; Grades 5–6: AOR = 1.60, 95% CI: 1.08–2.38, *p* = 0.020), better perceived academic performance (AOR = 1.76, 95% CI: 1.23–2.51, *p* = 0.002), higher happiness indexes for schools (AOR = 1.67, 95% CI: 1.03–2.71, *p* = 0.039), and studying in the DSS or private school (AOR = 1.50, 95% CI: 1.02–2.21, *p* = 0.42), remained significantly correlated with greater online learning effectiveness. Moreover, students who felt happy at school while less happy at home (AOR = 0.55, 95% CI: 0.33–0.93, *p* = 0.025) remained less likely to perceive online learning effectiveness than those who felt happy both at school and home (Table 3).

### Preference: Online Learning vs. In-Person Schooling

In the univariate model, preference in online learning was significantly related to a higher grade and self-perceived loneliness. Students who felt less happy both at the school and home and those who felt happy at home while less happy at school were also significantly related to the preference in online learning than those who felt happy both at school and home. However, sufficient sleep, better perceived academic performance, no intention of school transfer, higher happiness indexes for schools, and being satisfied with parents and self-performance were related to the less likelihood of preference in online learning (*p* < 0.05, Table 2).

In the multiple model, higher grade (Grades 3–4: AOR = 4.74, 95% CI: 2.20–10.22, *p* < 0.001; Grades 5–6: AOR = 3.77, 95% CI: 1.70–8.36, *p* = 0.001) and more teacher–student interaction during online learning (AOR = 2.32, 95% CI: 1.17–4.61, *p* = 0.017) remained positively related to the preference in online learning. Students who felt happy at home while less happy at school (AOR = 5.20, 95% CI: 2.19–12.34, *p* < 0.001) were still more likely to prefer online learning than those who felt happy both at the school and home. Students who were satisfied with their parents (AOR = 0.53, 95% CI: 0.29–0.95, *p* = 0.033) remained less likely to prefer online learning (Table 3).

## Discussion

Overall, in-person schooling was still the preferred learning mode among primary school students. During COVID-19 pandemic, less than three-fifths of the students were satisfied with their schools’ online learning arrangement and only nearly half regarded it as effective. Moreover, the grade of study, school type, happiness at school and home, satisfaction with parents, perceived academic performance, teacher–student interaction during online learning, and school’s happiness index were associated with the primary school Students’ satisfaction, perceived effectiveness, or preference in online learning.

Teacher–student interaction was of notable importance, which echoed prior research findings among university students. A study found that lack of interaction with instructors was a significant challenge perceived by college students (Adnan and Anwar, 2020). Also, Students’ attention declined quickly, especially when they were lack of reminders from teachers or peers (Bradbury, 2016) and encountered external stimuli that could distract them from studying (Wilson, 2004). The teacher–student interaction could facilitate more satisfaction and better learning outcomes in online learning (Alqurashi, 2019) by capturing the Students’ attention, guiding them to be focused during online learning, and obtaining Students’ constant and instant feedback for teachers to adjust teaching methods (Baber, 2020).

The grade of study was associated with Students’ perceived effectiveness and preference in online learning: younger primary students reflected more negative attitudes. They probably lacked the self-confidence to use digital platforms critically for learning (Drane et al., 2020) and still need to improve their self-regulation and attention skills (Gallagher and Cottingham, 2020). Thus, they would rely more on cognitive scaffolding (Tomasik et al., 2020). Parents and teachers may also need to help younger children with technical problems (Putri et al., 2020), guide them to be focused (Lau and Lee, 2020), and explain more to facilitate their understanding. Furthermore, adaptability could be another concern among grades 1–2 students who had engaged in a new school environment for only a short time. Adaptability was substantiated as a direct predictor of positive (persistence, planning, and task management) and negative (disengagement and self-handicapping) behavioral engagement (Collie et al., 2017). In this way, grades 1–2 students with lower adaptability skills may present weaker engagement during online learning, which further affected their online learning experiences and preferences passively.

The perceived academic performance was significantly correlated with the perceived effectiveness in online learning. As the academic performance was influenced by self-efficacy [ones’ beliefs that one can successfully perform given academic tasks at designated levels (Schunk, 1991)] (Alivernini and Lucidi, 2011), students having greater academic self-efficacy tended to attain better academic performance. So, those who perceived better academic performance could have higher self-efficacy in online learning, which resulted in their greater perceived effectiveness in online learning.

Happiness was a significant factor associated with various online learning outcomes. Students who reported lower happiness levels at school preferred online learning. It was suggested that children with unhappy school experiences (Murray-Harvey and Slee, 2010) tended to exhibit a variety of adaptive difficulties, such as academic concerns and interpersonal relationship obstacles (Whitley et al., 2012). These students would thrive in isolated distant learning, which could protect them from the pressure to look good, socialization, and bullying at school (Kaden, 2020). However, lower happiness levels at home could weaken Students’ satisfaction and perceived effectiveness in online learning. A lower happiness level at home can result from child abuse and domestic violence (Sternberg et al., 1993), unpleasant relationships with parents or siblings (Pike et al., 2005), and restricted personal space (Foye, 2017). Successful online learning required the capability of dealing with interruptions and emergencies and then refocus, which was harder to achieve while learning at home (McSporran and Young, 2001), especially for children exposed to domestic violence and quarrels. So, students with unhappy experiences at home could dislike online learning and hardly gain a positive online learning outcome. Also, this study found that students being satisfied with their parents would weaken their preference for online learning. These children receiving high parental care and support were more robust and more competent in tackling challenges (Parker and Benson, 2004; Trumpeter et al., 2008), thus would prefer to attend school in person. By contrast, having a child with school refusal, a parent may protect the child from unpleasant experiences and allow the child to depend excessively on them (Christogiorgos and Giannakopoulos, 2014), which could stimulate the child’s preference for learning at home. Additionally, higher happiness indexes for the school were correlated to Students’ satisfaction and perceived effectiveness in online learning. A Happy Schools Framework was proposed constituting 22 criteria of three related categories: (1) people, regarding social relationships; (2) process, regarding teaching and learning methods; and (3) place, regarding contextual factors (Salmon, 2016). Happier schools could provide more facilities and deliver diverse teaching methods, fostering interpersonal relationships and creating a better environment for Students’ learning and play. So, when online learning was necessarily applied, these schools could deliver more satisfying online learning experiences for students. The DSS and private schools delivered online education more effectively, as reported by students. These schools were more flexible in curriculum design and resources allocation than aided schools (Chiu and Walker, 2007), so they were more likely to apply student-centered methods in teaching. However, whether this association exists requires further study to verify.

### Strengths and Limitations

This study reflected the primary school Students’ attitudes toward online learning during COVID-19 pandemic, in which the population is much less studied than older students. This study explored potential factors from family, school, and individual aspects that could influence the Students’ attitudes. The survey quality was robust through a pilot study, elaborated survey design (i.e., audios, pictures, and emojis), and anonymization.

However, some limitations existed. First, a convenient sample was recruited due to COVID-19, so we should be conservative in generalizing the study findings. The rates shown in this study might have reflected a higher rate in the Students’ online learning satisfaction and perceived effectiveness, as the schools with better online education arrangements and learning outcomes would be more willing to participate in this study. However, the results of associated factor analyses should be acceptable to reflect the actual situation as the study sample was relatively large, with students across all six grades from different types of schools. Second, short questions were self-developed, as long surveys were undesirable for younger students. Some other factors were not explored, such as details of online learning arrangement (e.g., learning contents and class duration) and environment and facilities at home (e.g., access to technological devices and Internet, learning space, and disturbances from family members). Third, some degree of effect on the result may be derived from the extra cognitive scaffolding (audios, pictures, and emojis) provided to grades 1–3 students. However, this was necessary to ensure they gained equivalent comprehension on the questionnaire as grades 4–6 students and inevitable in studying younger children. Nevertheless, an expert panel was established, and a pilot study was conducted to guarantee the acceptability and feasibility of the questionnaire.

### Implications and Further Research

This study provides substantial evidence for better design and further research on children’s online learning. First, meaningful teacher–student interactions are essential in online learning practice. Schools and teachers should develop effective communication channels, e.g., discussion boards, individual reviews, emails, and polls, to understand Students’ needs, preferences, and learning progress. Teachers should be equipped with advanced pedagogical skills and devise more interactive activities to engage students in online classes. Periodic feedback on tests should also be provided (Alamri and Tyler-Wood, 2017; Baticulon et al., 2021). Second, extra attention, encouragement, and acceptance of errors (Twomey, 2006) should be given to younger primary students and those with poor academic performance. A blended learning mode might be more helpful for younger children when constant face-to-face learning was impossible (Musgrove and Musgrove, 2004). For their short attention spans, teachers might limit learning activities to 15–20 min at the beginning of an academic year and gradually increase subsequently (Schunk, 2012). Furthermore, workshops and activities should be delivered to enhance Students’ self-learning skills and self-efficacy in online learning. Third, a healthy and pleasant home-schooling environment is paramount in online learning. Parents should provide supportive company, stable internet access, and sufficient learning space. Independent skills, solitude, and computer self-efficacy (Jan, 2015) need to be emphasized to cultivate the children’s adaptability. Fourth, it is important to incentivize happiness culture and build up happy schools in the long run. Early childhood education serves as a foundation of immense value for children’s future development. Schools should gain a deeper insight into how children perceive they live and study in a happy academic environment. Teachers can regularly talk with students to understand their school experience. The government may consider investing more in educational research to practically develop happier schools from people, process and place aspects (Salmon, 2016).

For research implications, qualitative studies among students and their stakeholders (e.g., parents, teachers, and policymakers) are encouraged to investigate how the identified factors in this study have influenced Students’ online learning. Further studies are also warranted to explore other potential factors such as teaching design and family issues and follow-up the students longitudinally to understand the associations suggested in this study.

## Data Availability Statement

The original contributions presented in the study are included in the article/supplementary material, further inquiries can be directed to the corresponding author/s.

## Ethics Statement

The studies involving human participants were reviewed and approved by the Survey and Behavioral Research Ethics Committee, The Chinese University of Hong Kong. Written informed consent to participate in this study was provided by the participants’ legal guardian/next of kin.

## Author Contributions

XZ: formal analysis, data curation, and writing—original draft. DZ: conceptualization, methodology, investigation, writing—original draft, writing—review and editing, supervision, project administration, and funding acquisition. EL: investigation, writing—review and editing, and project administration. ZX and EM: investigation, data curation, writing—review and editing, and project administration. ZZ, PM, and XY: writing—review and editing. SW: writing—review and editing, supervision, and funding acquisition. All authors contributed to the article and approved the submitted version of the manuscript.

## Conflict of Interest

The authors declare that the research was conducted in the absence of any commercial or financial relationships that could be construed as a potential conflict of interest.

## Publisher’s Note

All claims expressed in this article are solely those of the authors and do not necessarily represent those of their affiliated organizations, or those of the publisher, the editors and the reviewers. Any product that may be evaluated in this article, or claim that may be made by its manufacturer, is not guaranteed or endorsed by the publisher.

## Footnotes

1 There are four main types of schools in Hong Kong—government, aided, the direct subsidy scheme (DSS), and private schools. Government schools are fully funded by the government, who controls the schools’ hiring practice and student admission. Aided schools, largely subsidized by the government, can only charge small amount of school fees from students, and are responsible for the hiring practice on their own. The DSS schools, receiving government subsidies, can collect school fees up to a certain amount for the provision of additional support services and school facilities, and have greater flexibility in resources deployment, curriculum design, student admission, etc. Private schools do not receive any government subsidy and have the greatest organizational freedom (Chiu and Walker, 2007; Bray et al., 2014) (Education Bureau).
